# Epidermal autophagy and beclin 1 regulator 1 and loricrin: a paradigm shift in the prognostication and stratification of the American Joint Committee on Cancer stage I melanomas†

**DOI:** 10.1111/bjd.18086

**Published:** 2019-06-19

**Authors:** R. Ellis, D. Tang, B. Nasr, A. Greenwood, A. McConnell, M.E. Anagnostou, M. Elias, S. Verykiou, D. Bajwa, T. Ewen, N.J. Reynolds, P. Barrett, E. Carling, G. Watson, J. Armstrong, A.J. Allen, S. Horswell, M. Labus, P.E. Lovat

**Affiliations:** ^1^ Institute of Cellular Medicine Newcastle University Newcastle upon Tyne U.K.; ^2^ Department of Dermatology James Cook University Hospital Middlesbrough U.K.; ^3^ Department of Pathology University of North Durham Hospital Durham U.K.; ^4^ Department of Pathology James Cook University Hospital Middlesbrough U.K.; ^5^ Department of Pathology St James's University Hospital Leeds U.K.; ^6^ Faculty of Health Sciences and Wellbeing University of Sunderland Sunderland U.K.; ^7^ NIHR Newcastle In Vitro Diagnostics Co‐operative Newcastle University Newcastle upon Tyne U.K.; ^8^ Bioinformatics and Bio Statistics Group The Francis Crick Institute London U.K.

## Abstract

**Background:**

The updated American Joint Committee on Cancer (AJCC) staging criteria for melanoma remain unable to identify high‐risk stage I tumour subsets.

**Objectives:**

To determine the utility of epidermal autophagy and beclin 1 regulator 1 (AMBRA1)/loricrin (AMLo) expression as a prognostic biomarker for AJCC stage I cutaneous melanoma.

**Methods:**

Peritumoral AMBRA1 expression was evaluated in a retrospective discovery cohort of 76 AJCC stage I melanomas. AMLo expression was correlated with clinical outcomes up to 12 years in two independent powered, retrospective validation and qualification cohorts comprising 379 AJCC stage I melanomas.

**Results:**

Decreased AMBRA1 expression in the epidermis overlying primary melanomas in a discovery cohort of 76 AJCC stage I tumours was associated with a 7‐year disease‐free survival (DFS) rate of 81·5% vs. 100% survival with maintained AMBRA1 (*P* < 0·081). Following an immunohistochemistry protocol for semi‐quantitative analysis of AMLo, analysis was undertaken in validation (*n* = 218) and qualification cohorts (*n* = 161) of AJCC stage I melanomas. Combined cohort analysis revealed a DFS rate of 98·3% in the AMLo low‐risk group (*n* = 239) vs. 85·4% in the AMLo high‐risk cohort (*n* = 140; *P* < 0·001). Subcohort multivariate analysis revealed that an AMLo hazard ratio (HR) of 4·04 [95% confidence interval (CI) 1·69–9·66; *P* = 0·002] is a stronger predictor of DFS than Breslow depth (HR 2·97, 95% CI 0·93–9·56; *P* = 0·068) in stage IB patients.

**Conclusions:**

Loss of AMLo expression in the epidermis overlying primary AJCC stage I melanomas identifies high‐risk tumour subsets independently of Breslow depth.

**What's already known about this topic?**

- There is an unmet clinical need for biomarkers of early‐stage melanoma.
- Autophagy and beclin 1 regulator 1 (AMBRA1) is a proautophagy regulatory protein with known roles in cell proliferation and differentiation, and is a known tumour suppressor.
- Loricrin is a marker of epidermal terminal differentiation.

**What does this study add?**

- AMBRA1 has a functional role in keratinocyte/epidermal proliferation and differentiation.
- The combined decrease/loss of peritumoral AMBRA1 and loricrin is associated with a significantly increased risk of metastatic spread in American Joint Committee on Cancer (AJCC) stage I tumours vs. melanomas, in which peritumoral AMBRA1 and loricrin are maintained, independently of Breslow depth.

**What is the translational message?**

- The integration of peritumoral epidermal AMBRA1/loricrin biomarker expression into melanoma care guidelines will facilitate more accurate, personalized risk stratification for patients with AJCC stage I melanomas, thereby facilitating stratification for appropriate follow‐up and informing postdiagnostic investigations, including sentinel lymph node biopsy, ultimately resulting in improved disease outcomes and rationalization of healthcare costs.

## Abstract 2

https://doi.org/10.1111/bjd.18658 available online

**Linked Editorial:** https://doi.org/10.1111/bjd.18555

Melanoma is one of the most devastating skin cancers, with a worldwide incidence that continues to rise.^1^, ^2^ Although the introduction of targeted and immune therapies has revolutionized treatment of metastatic disease, the largest proportion of patients presenting to clinicians have thin, early‐stage melanomas yet to benefit from therapeutic innovation, in part related to a lack of credible biomarkers of disease progression.

Currently, disease staging and risk prediction is based on histological characterization of the primary tumour, including depth of tumour invasion (Breslow depth) and the presence of epidermal ulceration, forming the basis of the seventh edition of the American Joint Committee on Cancer (AJCC) staging criteria.^3^ The recently updated eighth edition of the AJCC guidelines came into effect in January 2018,^4^ with removal of mitotic count and reduction of Breslow depth for stage IA melanomas to 0·8 mm. However, these criteria are still unable to identify reliably which individuals with seemingly low‐risk, early melanomas are at specific risk of disease progression, occurring in up to 15% of patients with AJCC stage I melanoma.^4^, ^5^ An urgent unmet need for credible prognostic biomarkers able to identify patients with high‐risk, early‐stage melanomas, facilitating appropriate counselling and follow‐up [including guidance on the need for sentinel lymph node biopsy (SLNB)] or access to clinical trials and potentially adjuvant systemic treatment,^6^, ^7^ thus remains.

We have identified two protein markers, autophagy and beclin 1 regulator 1 (AMBRA1) and loricrin, in the epidermis overlying primary melanomas, whose expression is lost in high‐risk AJCC stage I melanomas, but which are retained over genuinely low‐risk tumours. The role of AMBRA1 (a proautophagy regulatory protein) in melanoma progression was initially evaluated by immunohistochemistry (IHC) in a retrospective melanoma discovery cohort following previous reports of the role of autophagy in melanomagenesis;^8^, ^9^ however, unlike previously investigated autophagy biomarkers such as p62,^8^ AMBRA1 revealed variations in expression within the epidermis overlying primary melanomas, rather than within the tumour itself, suggesting a specific role for this protein in epidermal differentiation.^9^ To investigate peritumoral AMBRA1 as a potential prognostic marker, we initially evaluated immunohistochemical semi‐quantitative expression in a retrospective discovery cohort of AJCC stage I melanomas. However, although loss of peritumoral AMBRA1 expression correlated with disease progression with an assay sensitivity of 100%, the relatively low assay specificity (33%) hindered its clinical utility. Subsequently, to improve specificity, peritumoral epidermal AMBRA1 expression was assessed in combination with epidermal loricrin (as a marker of keratinocyte terminal differentiation), with the primary aim of this study to validate combined peritumoral AMBRA1 and loricrin (AMLo) expression as a prognostic biomarker for early‐stage melanoma.

## Materials and methods

### Study design

#### Patient cohort selection

**Figure 1:**
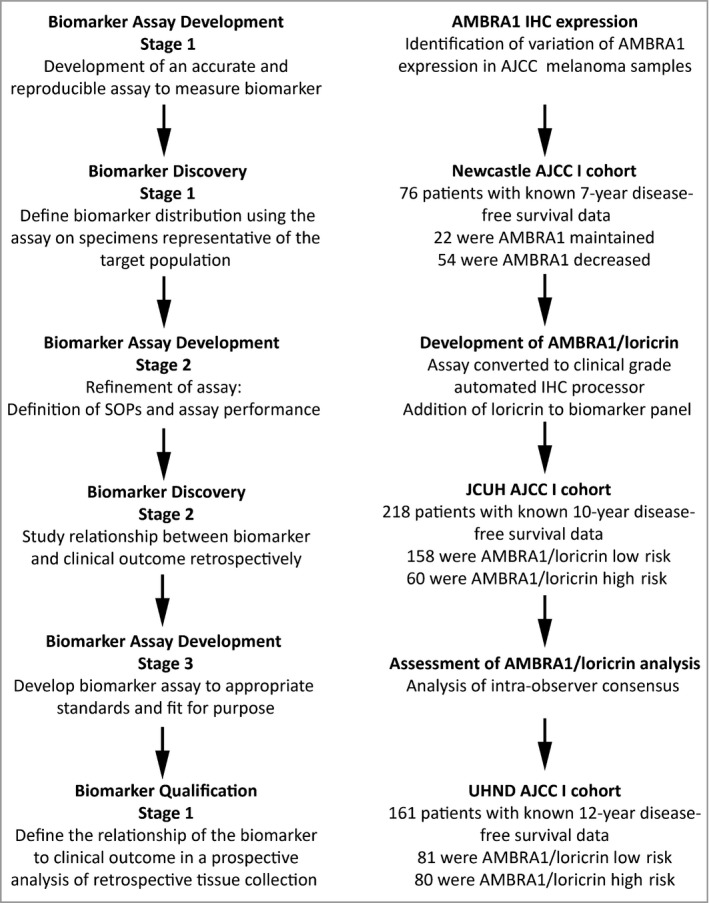
Study design followed the Cancer Research UK prognostic biomarker roadmap.^10^ Following initial identification of varied autophagy and beclin 1 regulator 1 (AMBRA1) epidermal expression in formalin‐fixed, paraffin‐embedded American Joint Committee on Cancer (AJCC; 2009) stage I and II primary cutaneous melanoma, an immunohistochemistry (IHC) assay was developed for initial assessment in a retrospective cohort of 76 patients with AJCC stage I melanoma derived from the Newcastle Hospital National Health Service Foundation Trust. Following validation and conversion of the assay to a fully automated IHC system, and the addition of loricrin, two further retrospective cohorts of 218 and 161 patients, respectively, with AJCC (2009) stage I melanomas were analysed for AMBRA1/loricrin expression levels and associated disease‐free survival data. SOP, standard operating procedure.

This study included three independent, statistically powered retrospective cohorts of AJCC stage I melanomas defined by the *AJCC Cancer Staging Manual*, seventh edition (Fig. 1). All cohorts were accessed following research ethics committee permission (ref. 08/H0906/95+5_Lovat). All steps of biomarker development followed the Cancer Research UK Prognostic Biomarkers Roadmap and were reported in line with the REMARK (REporting recommendations for tumour MARKer prognostic studies) guidelines.^10^, ^11^ The initial discovery cohort of 76 patients was derived from Newcastle Hospitals National Health Service Foundation Trust (NUTH), with subsequent discovery stage 2 (validation) and qualification retrospective cohorts derived from James Cook University Hospital (JCUH; *n* = 218) and University Hospital of North Durham, respectively (UHND; *n* = 161). Full patient selection and cohort demographics are described in Figure S1 (see Supporting Information).

#### Semi‐quantitative immunohistochemistry analysis of autophagy and beclin 1 regulator 1 and loricrin

Formalin‐fixed, paraffin‐embedded tissue sections (5 μm) were derived from primary melanomas from each cohort. The IHC methodology for AMBRA1 (Abcam, Cambridge, U.K.), loricrin (Abcam) and cytokeratin 5 (Novocastra; Leica Biosystems, Milton Keynes, U.K.) are detailed in Appendix S1 (see Supporting Information).

Semi‐quantitative analysis of epidermal AMBRA1 expression was undertaken using Leica Digital Image Hub software (Leica Biosystems). Up to 10 representative ×200 microscope fields were analysed for mean positive pixel intensity of AMBRA1 expression levels and compared to the mean AMBRA1 expression in the epidermis directly above the melanoma, allowing a relative percentage expression change to be calculated with the normal epidermis considered as 100% expression.

#### Validation of autophagy and beclin 1 regulator 1 and loricrin scoring

Visual AMBRA1 and loricrin scoring for all cohorts was undertaken by three individuals blinded to outcomes (R.E., E.C., G.W.); consensus agreement was reached for all samples. The ‘by‐eye’ analysis was grouped as either ‘maintained AMBRA1’ or ‘decreased/lost AMBRA1’. ‘Maintained AMBRA1’ was defined as no discernible difference in AMBRA1 expression between normal and peritumoral epidermis. ‘Decrease/lost AMBRA1’ was defined as *any* decrease or complete loss of AMBRA1 expression between normal and peritumoral epidermis.

All loricrin expression analysis was undertaken by eye, and loss of expression defined as any discernible break in the continuity of expression in the stratum corneum (Fig. S2; see Supporting Information).

### Statistical analysis

Survival analyses were conducted using the R function coxph(). Univariate estimates presented are coefficients, with 95% confidence intervals (CIs), resulting from a Cox fit with only that covariate as a predictor, while multivariate estimates are the associated coefficient from a full additive model, including all reported covariates (in particular, we did not refit to exclude nonsignificant predictors and considered no interaction terms).

Survival curves were generated using the R function survfit() to present Kaplan–Meier curves for a univariate model based on AMLo status, with presented *P*‐values as the result of a log‐rank (score) test for the associated univariate Cox model.

Statistical analysis for disease‐free survival (DFS) was planned for each cohort independently, as well as in combination for the JCUH and UHND cohorts. Further subcohort analysis was undertaken in AJCC stage IA and IB patients, as well as patients eligible for SLNB under the current UK National Institute for Health and Care Excellence guidelines (i.e. with a Breslow depth > 1 mm).

For the analysis of SLNB datasets, post‐test odds were used to calculate the positive and negative predictive values (i.e. clinically relevant measures of diagnostic accuracy). These are calculated by multiplying the pretest probability of a positive (negative) result (the prevalence or 1 – prevalence) with the diagnostic likelihood ratio of a positive (negative) test.^12^

## Results

### Peritumoral autophagy and beclin 1 regulator 1 expression and disease‐free survival in the Newcastle Hospitals National Health Service Foundation Trust discovery cohort

**Figure 2:**
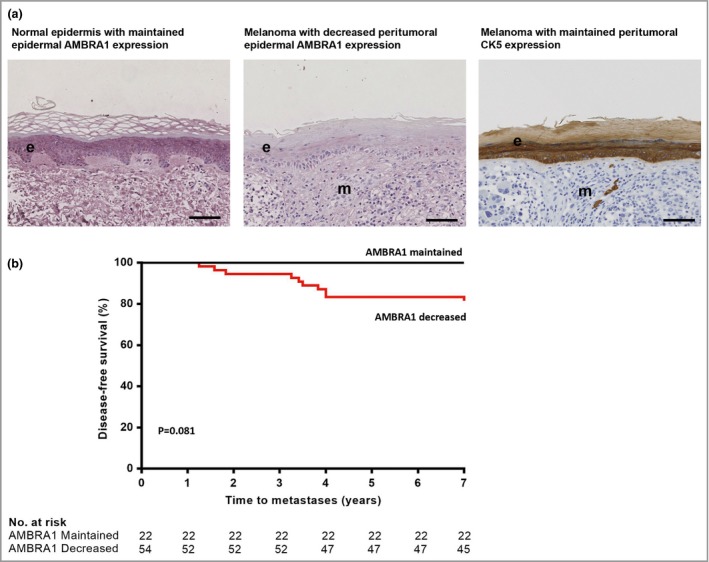
Relationship between peritumoral autophagy and beclin 1 regulator 1 (AMBRA1) expression and disease‐free survival in the Newcastle discovery American Joint Committee on Cancer (AJCC) stage I melanoma cohort. (a) Representative photomicrographs of immunohistochemical AMBRA1 (pink) or cytokeratin 5 (CK5; brown) staining in normal or matched peritumoral epidermis, where (e) represents the epidermis and (m) identifies melanoma (Breslow depth 1·2 mm, scale bars 100 μm). (b) AMBRA1 levels in the peritumoral epidermis of AJCC stage I melanomas were determined by visual inspection by a pathologist and defined as maintained or decreased. Estimated 7‐year disease‐free survival rates were determined with the Kaplan–Meier method and compared by a two‐sided log‐rank test (81·5% vs. 100%; *P* < 0·081).

To identify an association between epidermal AMBRA1 expression overlying primary tumours (peritumoral AMBRA1) and DFS in patients with AJCC stage I melanoma, 76 patients within the NUTH cohort were stratified as either having maintained or decreased AMBRA1 expression by visual analysis of peritumoral AMBRA1 expression (Fig. 2). All samples were also analysed for cytokeratin 5 (Fig. 2a), a pan‐epidermal marker, which revealed no association between the degree of melanoma epidermal invasion and peritumoral AMBRA1 expression.

Kaplan–Meier survival analysis revealed reduced 7‐year DFS in patients with decreased peritumoral epidermal AMBRA1 expression vs. 22 patients with maintained AMBRA1 expression [81·5% vs. 100% (*P* = 0·081); Fig. 2b]. However, the hazard ratio (HR) for disease recurrence among patients with decreased AMBRA1 expression vs. maintained expression could not be assessed owing to a lack of events in the maintained cohort.

Although decreased AMBRA1 expression was associated with a reduced 7‐year DFS with a sensitivity for identifying patients at risk of disease progression of 100%, the specificity of the test was only 33·3%, limiting clinical utility.

### Autophagy and beclin 1 regulator 1 as a marker of epidermal differentiation

To further underpin the role of AMBRA1 in epidermal differentiation, Western blot analysis for the expression of AMBRA1 and associated epidermal proteins was performed in primary keratinocytes undergoing calcium‐induced differentiation *in vitro*. Results revealed a consistent time‐dependent increase in AMBRA1 expression in line with increased differentiation; highlighted by decreased expression of cytokeratin 14 (a marker of basal keratinocytes) and increased loricrin expression. Conversely, small interfering RNA‐mediated knockdown of AMBRA1 in primary keratinocytes resulted in deregulated differentiation, as evidenced by downregulation of loricrin at both the mRNA and protein level (Fig. S3; see Supporting Information).

### Combined autophagy and beclin 1 regulator 1/loricrin peritumoral epidermal expression and disease‐free survival in the James Cook University Hospital and University Hospital of North Durham validation cohorts

**Figure 3:**
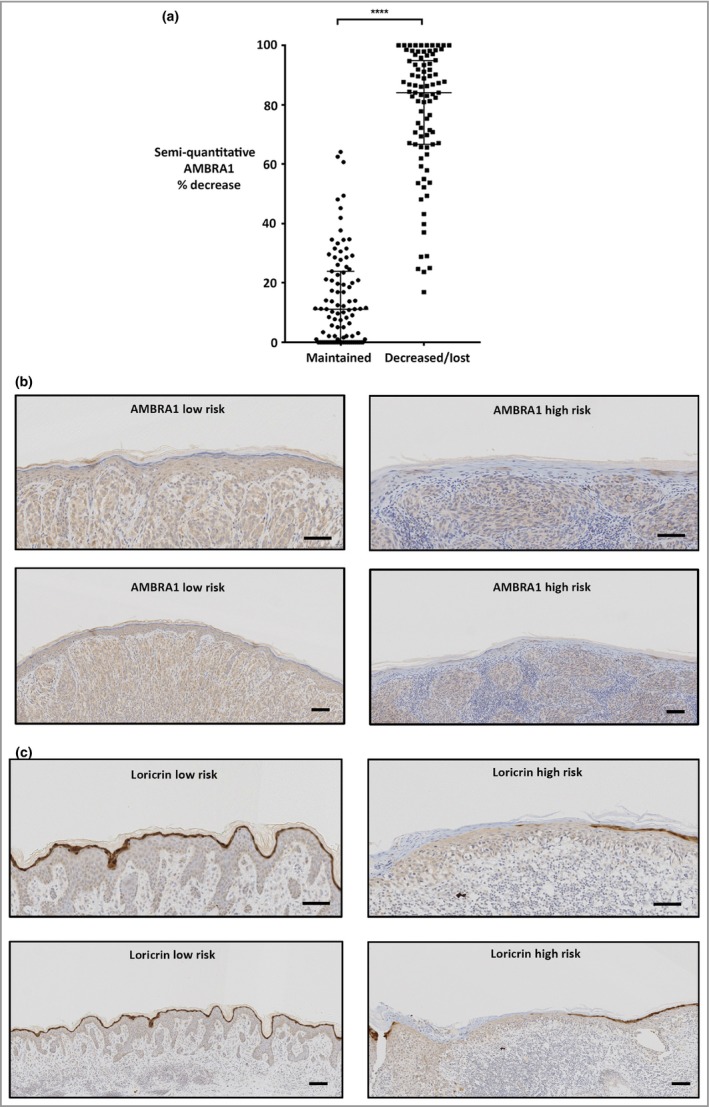
Immunohistochemistry analysis of autophagy and beclin 1 regulator 1 (AMBRA1) protein expression in the James Cook University Hospital American Joint Committee on Cancer stage I cohort. (a) Semi‐quantitative scoring of AMBRA1 (percentage decrease in peritumoral epidermis vs. normal epidermis at the section margins) vs. visual scoring of either maintained or decreased peritumoral AMBRA1 [horizontal bar indicates median with interquartile range (median 11·2% vs. 84·1%; Mann–Whitney, *P* < 0·001)]. (b) Representative photomicrographs of maintained (low risk) or decreased (high risk) immunohistochemical AMBRA1 staining in peritumoral epidermis at ×100 and ×200 magnification (scale bars 100 μm). (c) Representative photomicrographs of continuous (low risk) or broken (high risk) immunohistochemical loricrin staining in peritumoral epidermis at ×100 and ×200 magnification (scale bars 100 μm). ****indicates value is *P* < 0.00001.

To compare the method of determining AMBRA1 expression, both visual and semi‐quantitative analysis of peritumoral AMBRA1 expression were performed in the JCUH cohort (Fig. 3 and Fig. S4; see Supporting Information). The results revealed a significant difference in median percentage loss of AMBRA1 expression from 11·2% in the ‘no visual loss’ group to 84·1% in the ‘visual loss present’ group (Fig. 3b; Mann–Whitney, *P* <0·001). As such, any decrease or loss of peritumoral epidermal AMBRA1 expression when compared ‘by eye’ with the expression of AMBRA1 in normal epidermis was deemed as ‘high risk’ (Fig. S4). Furthermore, these observations confirmed the robustness of visual analysis, which was subsequently used alone for all other cohort analyses, and highlighted the major benefit of having an internal control (normal epidermis) present in each primary tumour excision sample.

Visual assessment of loricrin also defined two distinct subsets (Fig. 3b and Fig. S5; see Supporting Information).

**Figure 4:**
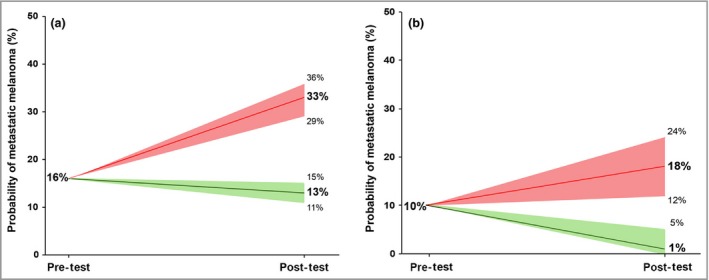
Relationship between peritumoral autophagy and beclin 1 regulator 1 (AMBRA1)/loricrin expression and disease‐free survival in sentinel lymph node biopsy (SLNB)‐eligible patients. (a) Post‐test probabilities after SLNB for metastatic melanoma in MSLT‐1 (Multicenter Selective Lymphadenectomy Trial) intermediate‐thickness cohort. (b) Post‐test probabilities after analysis of AMBRA1/loricrin expression in SLNB‐eligible American Joint Committee on Cancer stage I melanoma samples.

To confirm the association of decreased DFS with alterations of the peritumoral epidermis, IHC expression of loricrin was also assessed in a small subgroup of the NUTH discovery cohort of AJCC stage I melanomas, revealing an association between peritumoral loricrin loss and decreased DFS with a high degree of assay specificity but lower sensitivity (Fig. 4). However, strikingly, when epidermal loricrin expression was combined with epidermal AMBRA1 expression, the results revealed that decreased peritumoral expression of both markers was associated with 100% sensitivity and specificity for identifying truly high‐risk melanomas in this subcohort (Fig. S6; see Supporting Information).

Consequently, the combination of AMLo was deemed as ‘high risk’ if AMBRA1 peritumoral expression was decreased or lost, *and* if there was any apparent break in the continuous expression of epidermal loricrin.

Subsequent analysis of combined epidermal AMLo expression was undertaken in two further validation cohorts of AJCC stage I melanoma patients (JCUH and UHND cohorts). These cohorts were statistically powered to provide 80% and ~95% power, respectively, to detect an HR of > 4·0 (as per biomarker discovery cohort) at the *P* = 0·05 level, assuming equal group sizes and a representative number of metastatic events as expected in AJCC stage I melanomas (~10%).^3^ A detailed description of patient datasets for these analyses is provided in Figure S3 (see Supporting Information).

The IHC protocol was further refined from the discovery cohort as detailed in Appendix S1 (see Supporting Information), and undertaken on a fully automated clinical platform within the JCUH Pathology Department (Fig. 3b).

**Figure 5:**
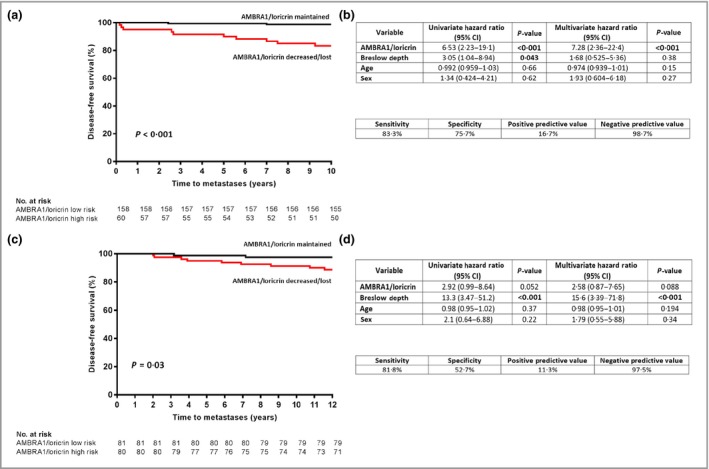
Relationship between peritumoral autophagy and beclin 1 regulator 1 (AMBRA1)/loricrin expression and disease‐free survival in the validation cohorts. AMBRA1 and loricrin levels in the peritumoral epidermis of American Joint Committee on Cancer stage I melanomas were determined by visual inspection by a pathologist and defined as maintained or decreased. Ten‐year disease‐free survival rates were determined with the Kaplan–Meier method and compared by a two‐sided log‐rank test in both the (a) James Cook University Hospital [JCUH; 84·8% vs. 98·1% (*P* < 0·001)] and (c) University Hospital of North Durham [UHND; 88·8% vs 97·5% (*P* = 0·033)] cohorts. Assay performance and univariate and multivariate cox regression analysis of disease‐free survival in the (b) JCUH and (d) UHND cohorts. CI, confidence interval.

Analysis of 10‐year DFS in the JCUH cohort of 218 AJCC stage I melanomas revealed reduced DFS in 60 patients stratified as AMLo high‐risk vs. 158 patients defined as AMLo low‐risk [83·3% vs. 98·7% (*P* = 0·001); Fig. 5a] with a multivariate HR for disease recurrence in patients with high‐risk AMLo expression of 7·28 [95% CI 2·36–22·41 (*P* < 0·001); Fig. 5b].

Analysis of DFS over 12 years in the 161 patients in the UHND cohort also demonstrated reduced DFS in 80 patients with high‐risk AMLo expression vs. 81 patients with low‐risk AMLo expression [88·8% vs. 97·5% (*P* = 0·03); Fig. 5c]. The multivariate HR for disease recurrence in patients with high‐risk AMLo expression in this cohort was 2·58 [95% CI 0·87–7·65 (*P* = 0·088); Fig. 5d].

**Figure 6:**
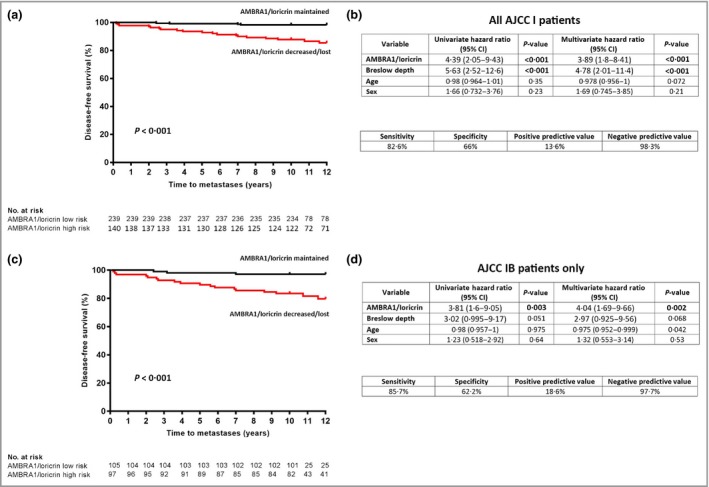
Relationship between peritumoral autophagy and beclin 1 regulator 1 (AMBRA1)/loricrin expression and disease‐free survival in the combined validation cohorts. AMBRA1 and loricrin levels in the peritumoral epidermis of American Joint Committee on Cancer (AJCC) stage I melanomas were determined by visual inspection by a pathologist and defined as maintained or decreased. Twelve‐year disease‐free survival (DFS) rates were determined with the Kaplan–Meier method and compared by a two‐sided log‐rank test in the combined validation cohort (86·1% vs. 97·9%; *P* < 0·001). (b) Assay performance and univariate and multivariate Cox regression analysis of DFS in the combined cohort. (c) Twelve‐year DFS rates were determined with the Kaplan–Meier method and compared by a two‐sided log‐rank test in AJCC stage IB melanomas of the combined validation cohort (80·5% vs. 96·2%; *P* < 0·001). (d) Assay performance and univariate and multivariate Cox regression analysis of DFS in AJCC stage IB melanomas of the combined cohort. CI, confidence interval.

By combining the two validation cohorts to increase the power to detect an effect of AMLo alone (Fig. 6), the results revealed a highly significant reduction in DFS in 140 patients with high‐risk AMLo expression compared with 239 patients with low‐risk AMLo expression [85·5% vs. 98·3%; (*P* < 0·001); Fig. 6a]. The multivariate HR for disease recurrence among patients with high‐risk AMLo expression was 3·89 [95% CI 1·8–8·41 (*P* < 0·001); Fig. 6b]. As a further guide to clinical utility, analysis of the combined cohort revealed a sensitivity of 82·6%, specificity of 66%, a positive predictive value of 13·6% and, most importantly, a negative predictive value of 98·3% (Fig. 6b).

Furthermore, subgroup analysis of the study combined cohort of stage IB (eighth edition AJCC) patient set (Fig. 6c) revealed a statistically stronger correlation between AMLo stratification and DFS. In this subcohort, DFS was 97·1% in the AMLo low‐risk group (*n* = 105) vs. only 79·6% in the AMLo high‐risk cohort [*n* = 97 (*P* < 0·001); Fig. 6c]. Multivariate analysis revealed an AMLo HR of 4·04 [95% CI 1·69–9·66 (*P* = 0·002); Fig. 6d], whereas there was less of a correlation between Breslow depth and overall DFS [multivariate analysis HR 2·97, 95% CI 0·93–9·56 (*P* = 0·068); Fig. 6d], suggesting that AMLo is a prognostic marker, independent of Breslow depth, which is able to stratify patient risk over and above AJCC staging alone.

## Discussion

We are currently experiencing what has been described as the ‘golden age’ of melanoma therapy,^13^ with an ever‐expanding arsenal of systemic medications available for patients with metastatic disease, resulting in increased survival periods beyond the expectations of the most optimistic of clinicians even 10 years ago.^14^, ^15^ The thrust of trials is now aimed at adjuvant initiation of these therapies, which has resulted in unprecedented results in patients with surgically resected, AJCC stage III melanomas at high risk of recurrence.^16^, ^17^ The natural progression to tackling melanoma finally is the initiation of systemic therapy at the earliest possible point at which high‐risk individuals can be identified, thus stopping the development of life‐threatening metastases or treating them before they have affected the patient's health.

As such, a yet‐unrealized dream for the management of melanoma is the ability to identify patients at the highest risk of progression as close to the time of diagnosis of a primary melanoma as possible, allowing increased surveillance and earlier therapeutic intervention. Conversely, patients at truly low risk of disease could be more confidently reassured and follow‐up regimes altered. The largest population of patients affected by melanoma have AJCC stage I disease. As such, any improvements in the care of this group of patients will affect the largest number of patients overall, with the associated potential economic benefits.

AJCC staging of melanoma alone, relying on Breslow depth and the presence of ulceration, is not a perfect predictor of outcome in the lowest‐risk, stage I group, where a small but significant proportion of patients will still die of their disease. Recent evidence‐based changes to the AJCC eighth edition staging criteria highlight a ‘breakpoint’ of 0·8 mm Breslow depth, with nonulcerated primaries below this depth classified as stage IA, with ulcerated tumours < 0·8 mm, or nonulcerated tumours from 0·8 to 2 mm Breslow depth, classified as stage IB. This has increased the number of patients now classified as having stage IB disease, with implications for increased surveillance of stage IB patients (5 years of follow‐up) vs. those with stage IA melanoma (1 year of follow‐up).

Herein we describe the discovery and validation of the combined expression of two protein biomarkers, AMBRA1 and loricrin, in the epidermis overlying primary AJCC stage I melanomas as a highly sensitive and specific prognostic biomarker. AMBRA1 is a proautophagy regulatory protein and our *in vitro* data further define a functional role for AMBRA1 in epidermal proliferation, and, like loricrin, in epidermal differentiation.

There is ongoing controversy about the clinical role of SLNB, with large‐scale trials raising questions about the prognostic and therapeutic role of SLNB, as well as highlighting the associated morbidity and healthcare costs of patients undergoing the procedure unnecessarily. As with melanoma in general, improved stratification of those patients at the highest risk of disease progression would potentially allow SLNB to further refine individual disease risk.

The Multicenter Selective Lymphadenectomy Trial (MSLT‐1) SLNB trial contained a cohort of 765 intermediate‐thickness primary tumours undergoing SLNB that ranged in Breslow depth from 1 to 3·5 mm.^18^ Overall, the pretest probability of a patient in this cohort developing a metastasis was 16%. In patients with a positive SLNB the number developing metastases was higher, giving a post‐test probability of developing metastases in this group of 33% (95% CI 29–36); however, a negative SLNB was still associated with a 13% chance of disease progression (95% CI 11–15%) (Fig. 4).^18^ Therefore, these data suggest that a positive SLNB is able to further risk‐stratify patients, yet a negative SLNB adds little to reassure patients about their true risk of metastasis.

Although analysis has been undertaken in separate cohorts, assessment of the outcomes in our SLNB‐eligible combined cohort (patients with a Breslow depth > 1 mm, as per U.K. guidelines) nevertheless revealed that a pretest probability of metastasis of 10% is increased to 18% (95% CI 12–24) in the AMLo high‐risk group. In contrast, low‐risk AMLo was associated with a post‐test probability of only a 1·4% chance of metastasis (95% CI 0–5; Fig. 4). In this context, these results highlight the potential of AMLo as a valuable pre‐SLNB test; identifying those patients that would receive no further benefit from SLNB, and increasing the positive predictive value of SLNB through use in only a high‐risk, AMLo refined cohort.

In summary, our study reveals AMLo as a novel prognostic biomarker for early‐stage cutaneous melanoma, over and above AJCC staging alone. This simple, IHC‐based marker will integrate seamlessly into standard clinical pathways of melanoma diagnostics, and allow the treating clinician a greater degree of certainty around disease outcomes. Given the current prevailing view that SLNB is a purely prognostic tool, and considering the cost and morbidity associated with SLNB, there is potential that the AMLo biomarker may later replace SLNB. Not only will this benefit the individual patient in terms of reduced psychological burden from greater clarity of disease risk, but it will also allow better healthcare resource utilization internationally. As the golden age of melanoma care continues, the adoption of the AMLo biomarker into clinical guidelines thus presents a major paradigm shift in melanoma prognostication and stratified personalized management for the future.

## Supporting information


**Fig S1.** Loss of autophagy and beclin 1 regulator 1 results in deregulated epidermal differentiation.

**Fig S2.** Epidermal expression of autophagy and beclin 1 regulator 1 and loricrin in normal skin and overlying benign naevi.

**Fig S3.** Patient demographics and selection pathways.

**Fig S4.** Scoring system for epidermal autophagy and beclin 1 regulator 1 expression.

**Fig S5.** Scoring system for epidermal loricrin expression.

**Fig S6.** Relationship between loricrin and autophagy and beclin 1 regulator 1 and loricrin expression in the Newcastle discovery cohort.

**Appendix S1.** Supplementary methods.
