# Supplementary figures and images for: Epidermal autophagy and beclin 1 regulator 1 and loricrin: a paradigm shift in the prognostication and stratification of the American Joint Committee on Cancer stage I melanomas
Source: Br J Dermatol. 2019 Jun 19;182(1):156–65. doi: 10.1111/bjd.18086 (PMC6973157; doi:10.1111/bjd.18086)

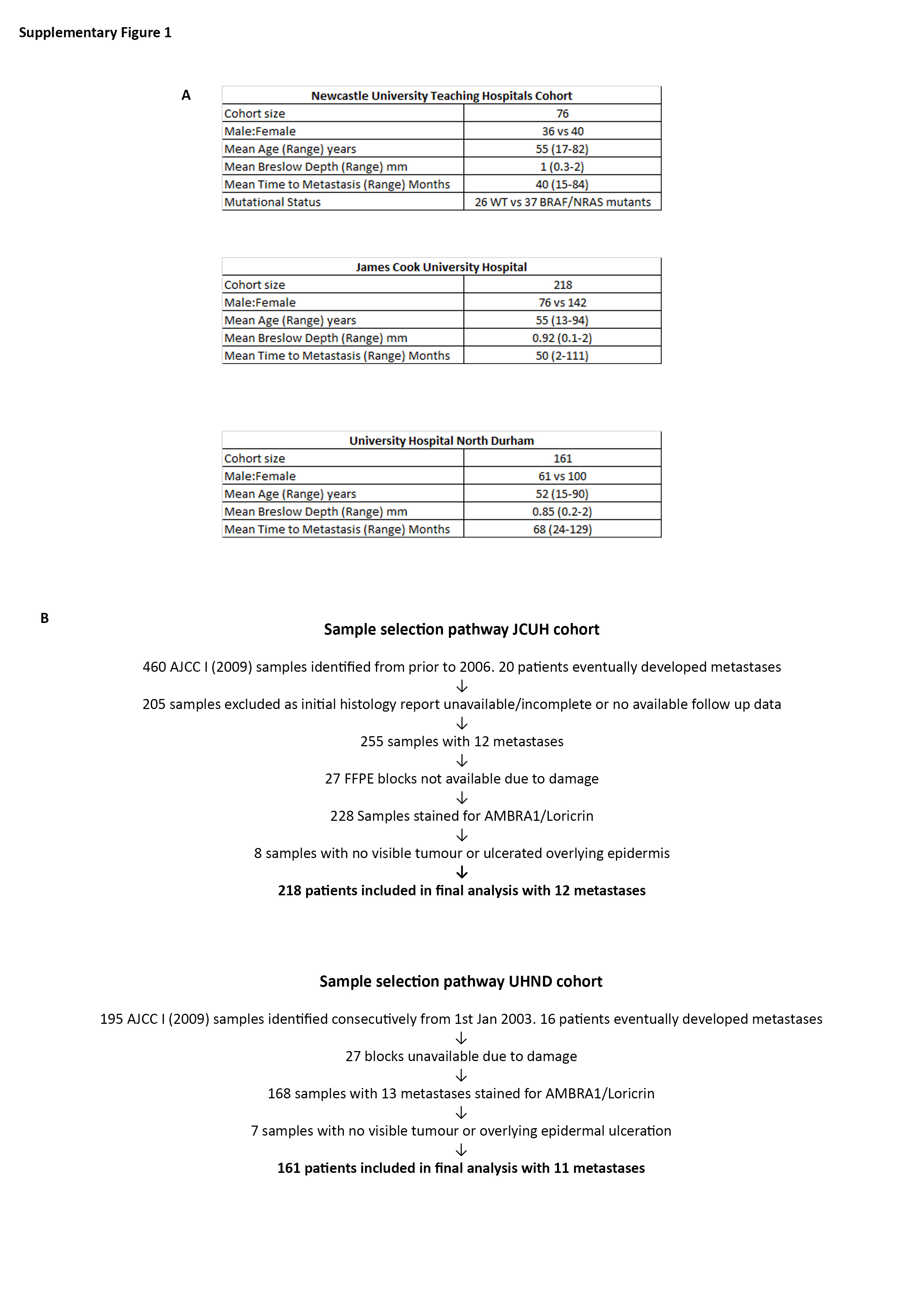

Supplement: Supplementary file 1 — Fig S1. Loss of autophagy and beclin 1 regulator 1 results in deregulated epidermal differentiation. [file BJD-182-156-s001.jpg]

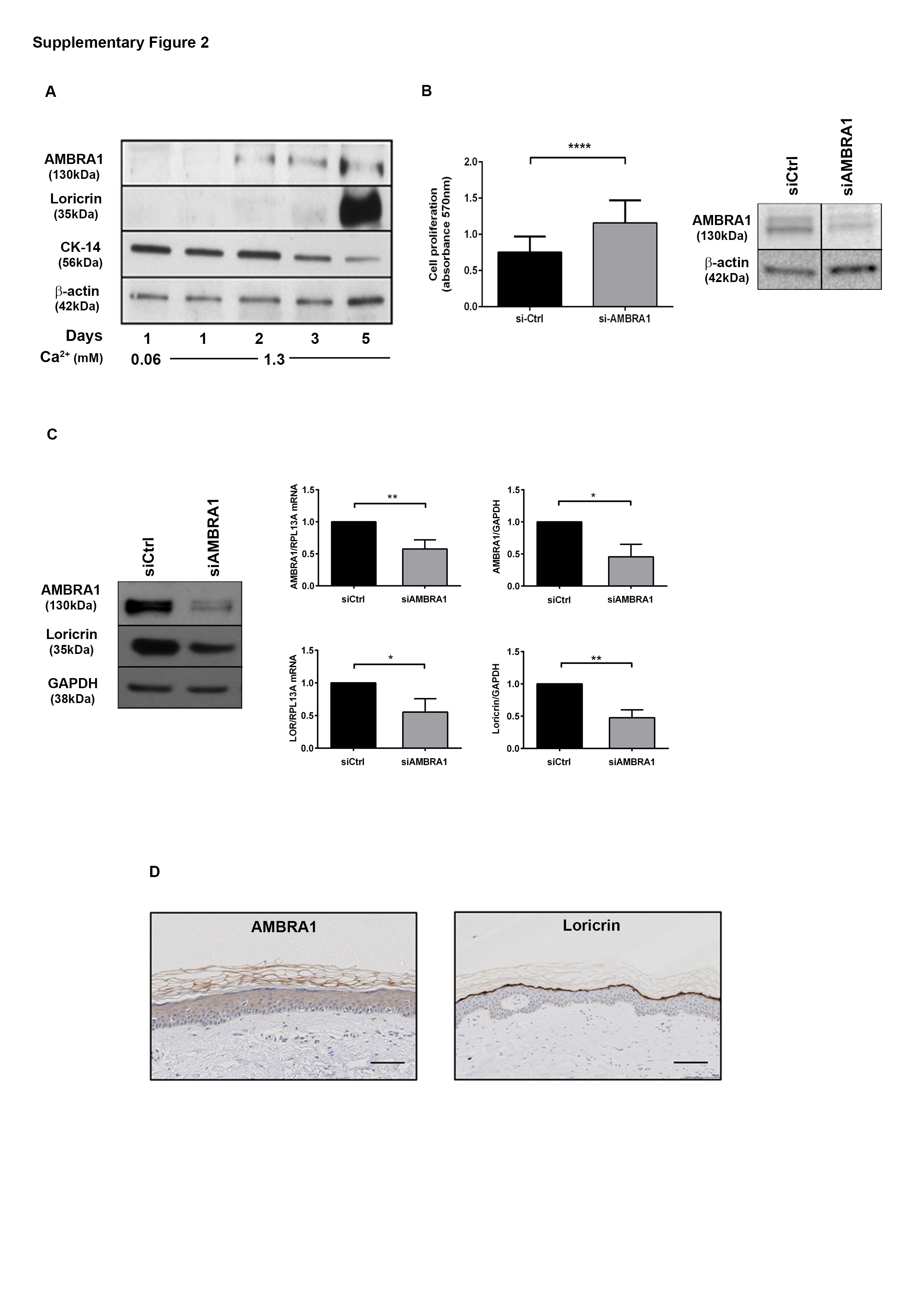

Supplement: Supplementary file 2 — Fig S2. Epidermal expression of autophagy and beclin 1 regulator 1 and loricrin in normal skin and overlying benign naevi. [file BJD-182-156-s002.jpg]

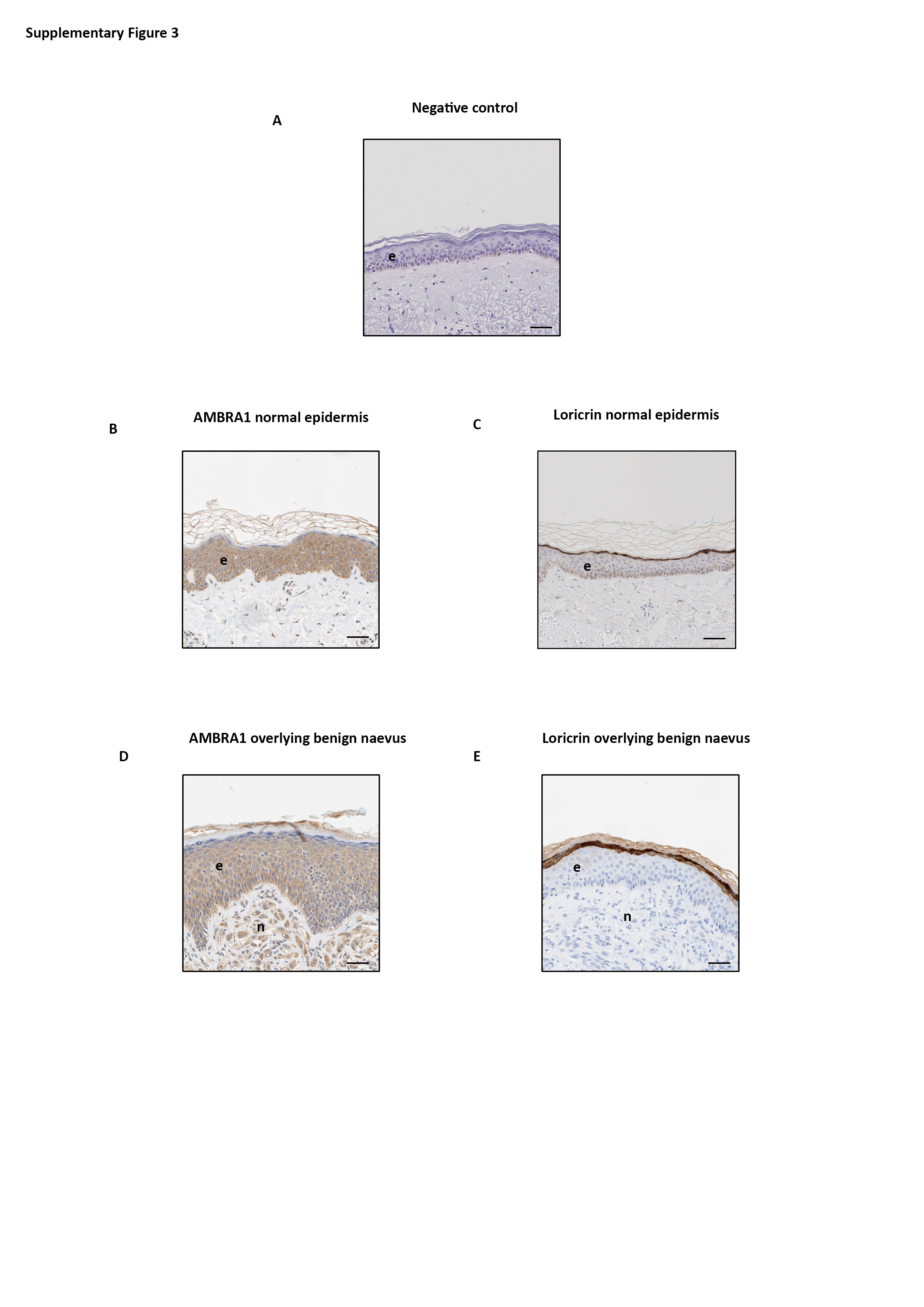

Supplement: Supplementary file 3 — Fig S3. Patient demographics and selection pathways. [file BJD-182-156-s003.jpg]

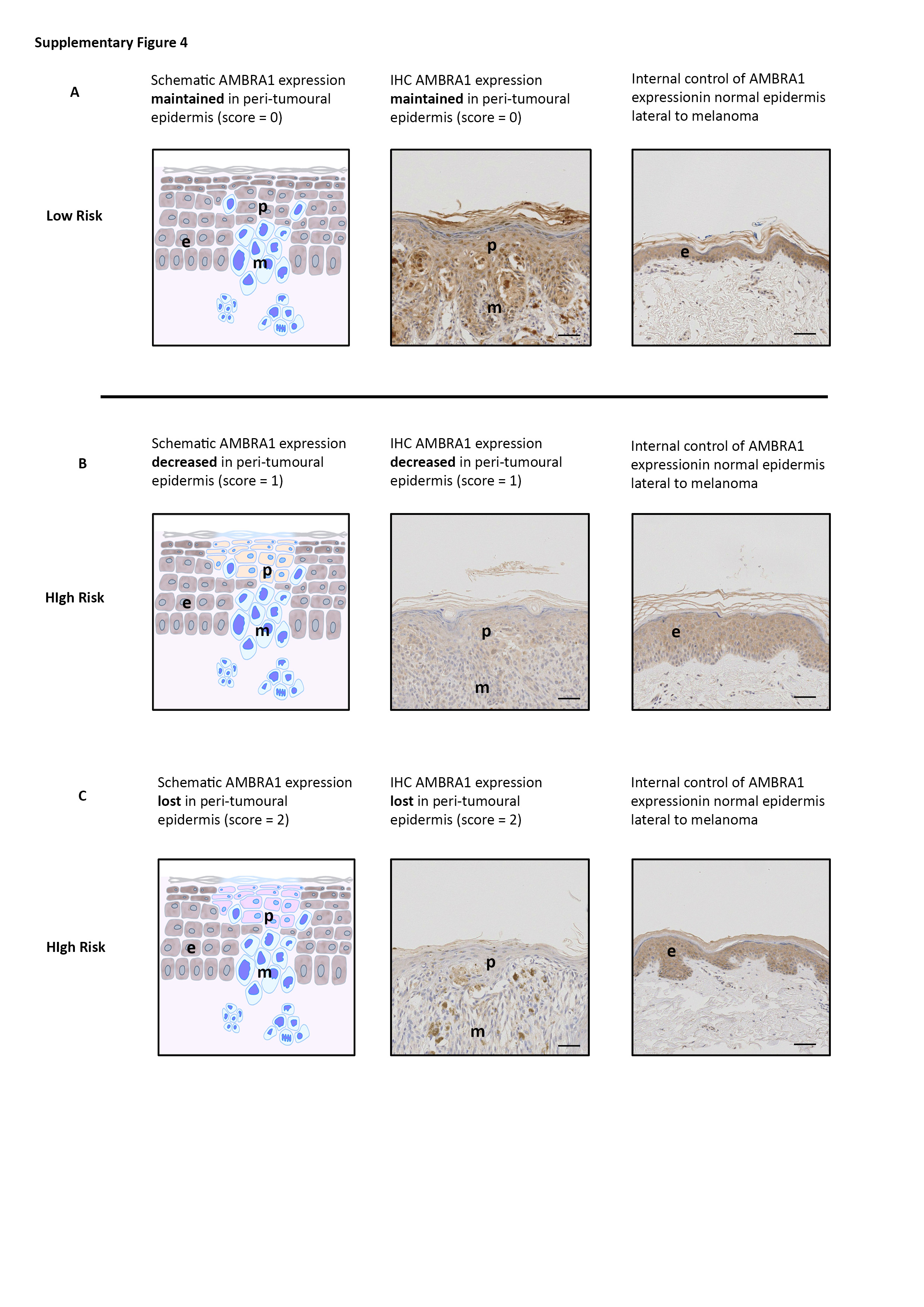

Supplement: Supplementary file 4 — Fig S4. Scoring system for epidermal autophagy and beclin 1 regulator 1 expression. [file BJD-182-156-s004.jpg]

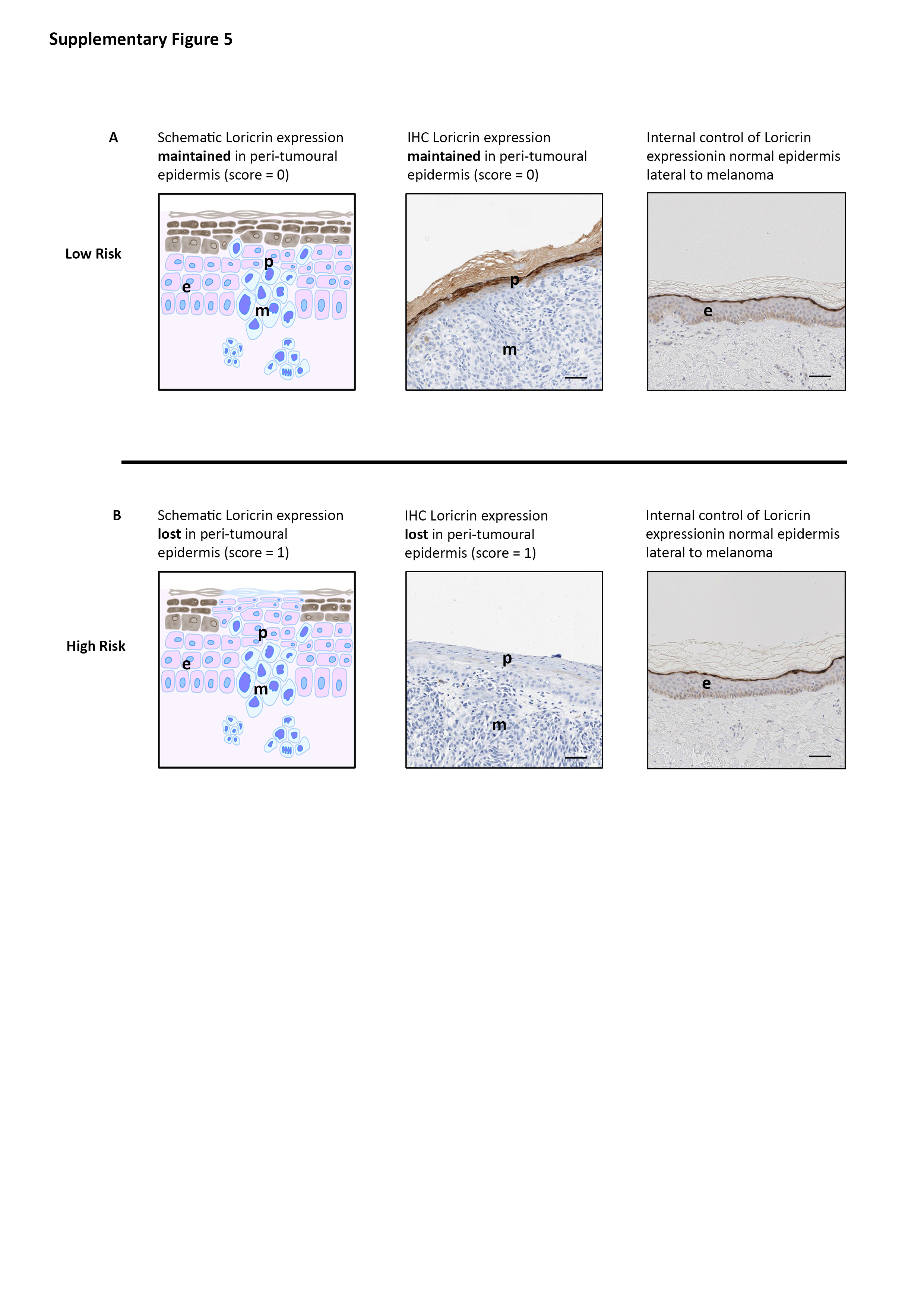

Supplement: Supplementary file 5 — Fig S5. Scoring system for epidermal loricrin expression. [file BJD-182-156-s005.jpg]

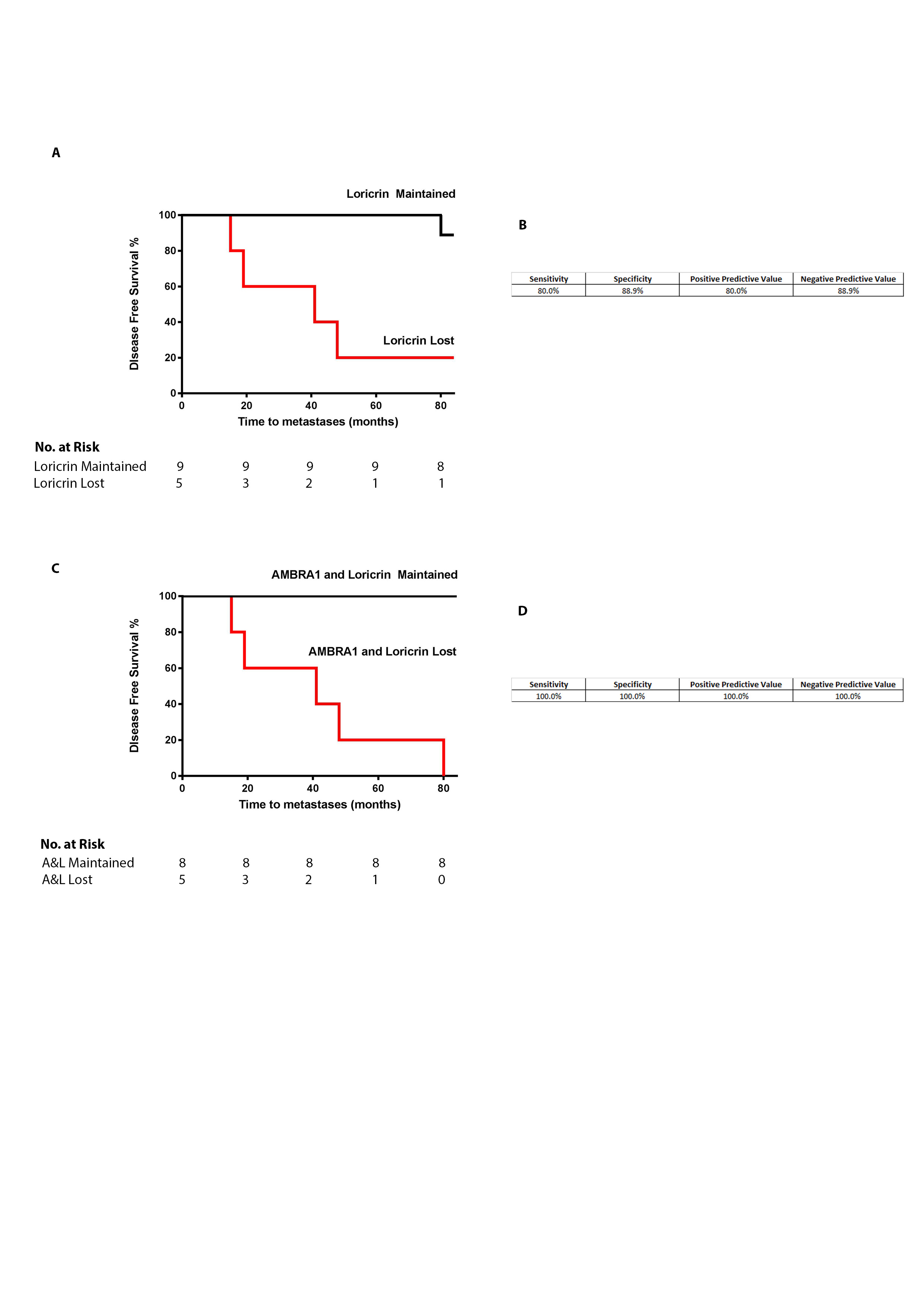

Supplement: Supplementary file 6 — Fig S6. Relationship between loricrin and autophagy and beclin 1 regulator 1 and loricrin expression in the Newcastle discovery cohort. [file BJD-182-156-s006.jpg]
