# Supplementary material for: Epidermal autophagy and beclin 1 regulator 1 and loricrin: a paradigm shift in the prognostication and stratification of the American Joint Committee on Cancer stage I melanomas
Source: Br J Dermatol. 2019 Jun 19;182(1):156–65. doi: 10.1111/bjd.18086 (PMC6973157; doi:10.1111/bjd.18086)
Supplement: Supplementary file 7 — Appendix S1. Supplementary methods. [file BJD-182-156-s007.docx]

**Supplementary Methods**

For manual detection of AMBRA1 expression tumours were subjected to pre-optimised IHC analysis (1) with antigen retrieval preformed in 10 mM Tris-Hcl (pH 7.6) and primary antibody binding detected with primary AMBRA1 (Abcam) diluted 1:200, visualised using VIP counterstaining (Vector Labs) .

Antigen retrieval conditions and antibody dilutions for the automated IHC detection of AMBRA1 (Abcam), Loricrin (Abcam) and Cytokeratin 5 (Novocastra) were optimised using a Ventana Benchmark XT automated IHC staining instrument (Ventana Medical Systems Inc.) with antibody binding visualised either with an Optiview DAB Detection Kit (Ventana Medical Systems Inc.) or an ultraView Universal DAB Detection Kit (Ventana Medical Systems Inc.), according to the manufacturers specifications.

Following visual validation of consistent AMBRA1 and Loricrin expression these antibodies were further analysed in the JCUH and UHND tissue cohort samples in the Pathology department of JCUH at concentrations of 1:300 AMBRA1, 1:1500 Loricrin, 1:100 Cytokeratin 5 with final counterstaining in haematoxylin for 8 minutes at room temperature. All IHC stained sections were digitally imaged using automated scanning of slides on a digital slide scanner (Leica SCN400) for subsequent visual and semi-quantitative analysis.

For utility analysis of each biomarker cohort the classification functions for sensitivity, specificity, positive predictive value and negative predictive value were undertaken:

Sensitivity = Number of true positive

Number of true positives + number false negatives

Specificity = Number of true negatives

Total number of well individuals in population

Positive Predictive Value = Number of true positives

Number of true positives + number of false positives

Negative Predictive Value = Number of false positives

Number of true negatives + number of false positives

For analysis of SLNB outcome data (Figure 6), analysis steps undertaken were:

LRp <- sensitivity/(1 - specificity)  Diagnostic likelihood ratio of a positive test

LRn <- (1 -sensitivity)/(specificity)  DLR n

PreTestOddsP <- prevalence/(1 - prevalence)  Pre test odds of positive result

PreTestOddsN <- (prevalence)/(1 -prevalence)  Pre test odds of negative result

PostTestOddsP <- PreTestOddsP*LRp   Post test odds of positive result PostTestOddsN <- PreTestOddsN*LRn  Post test odds of negative result

PostTestProbP <- PostTestOddsP/(PostTestOddsP + 1) – post test probability of positive result = PPV

PostTestProbN <- PostTestOddsN/(PostTestOddsN + 1)  post test probability of negative result  = 1- NPV

**References**

1. Ellis RA, Horswell S, Ness T, Lumsdon J, Tooze SA, Kirkham N, et al. Prognostic impact of p62 expression in cutaneous malignant melanoma. J Invest Dermatol. 2014;134(5):1476-8.
