# Supplementary material for: Epidermal autophagy and beclin 1 regulator 1 and loricrin: a paradigm shift in the prognostication and stratification of the American Joint Committee on Cancer stage I melanomas
Source: Br J Dermatol. 2019 Jun 19;182(1):156–65. doi: 10.1111/bjd.18086 (PMC6973157; doi:10.1111/bjd.18086)
Supplement: Supplementary file 8 [file BJD-182-156-s008.docx]

**Supplementary figure legends**

**Supplementary Figure 1. Loss of AMBRA1 results in deregulated epidermal differentiation.**

(A) Representative Western blot (n=3) for AMBRA1, loricrin, cytokeratin 14 (CK-14) and β-actin protein expression in primary keratinocytes following switch from culture in 0.06mM calcium chloride to culture in high calcium (1.3mM) for 5 days. (B) Cell proliferation (Sulphorhodamine B assay) or representative Western blot (n = 2) for AMBRA1 or β-actin protein expression in CCD-1106 keratinocytes following transfection with AMBRA1 siRNA (si-AMBRA1) or a non-targeting siRNA (si-Ctrl) by reverse transfection and culture for 7 days (for cell proliferation) or for 6 hours and subsequent culture for 5 days (for Western blot). (C) Western blot (n = 3) and RT-qPCR mRNA (n = 4) expression analysis of AMBRA1, Loricrin and GAPDH or RPL13A in primary keratinocytes transfected with control (si-Ctrl) or AMBRA1 (siAMBRA1) siRNA and incubated in high calcium (1.3 mM) for 5 days. Protein levels were quantified by densitometry, normalised to GAPDH, and presented relative to siCtrl (mean ± SD). mRNA expression levels were normalised to RPL13A and presented relative to siCtrl (mean ± SD). Unpaired one-sample T-test; *** P < 0.001, ** P < 0.01, * P< 0.05. (D) Representative IHC images of epidermal AMBRA1 and Loricrin expression in normal skin (Scale bar = 100µm).

**Supplementary Figure 2. Patient demographics and selection pathways**

(A) Demographic data of the Newcastle University Teaching Hospital, James Cook University Hospital and University Hospital of North Durham AJCC Stage I cohorts. (B) Sample selection pathway for the James Cook University Hospital and University Hospital of North Durham cohorts.

**Supplementary Figure 3. Epidermal expression of AMBRA1 and Loricrin in normal skin and overlying benign nevi.**

Representative photomicrographs of immunohistochemical negative control (A), AMBRA1 (B,D) or Loricrin staining (C,D) in the normal epidermis (A,B,C) as well as in the epidermis overlying benign melanocytic naevi (D, E). Scale bars = 100µm.

**Supplementary Figure 4. Scoring system for epidermal AMBRA1 expression.**

Schematic and representative IHC images of AMBRA1 expression peritumoural epidermis compared to matched normal epidermis at the section margins, showing maintained AMBRA1 (A; low risk, score = 0), decreased AMBRA1 (B; high risk, score = 1) and loss of AMBRA1 (C; high risk, score = 2) (m = melanoma, p = peri-tumoural epidermis, n = normal epidermis, scale bars = 100µm).

**Supplementary Figure 5. Scoring system for epidermal Loricrin expression.**

Schematic and representative IHC images of Loricrin expression peritumoural epidermis compared to matched normal epidermis at the section margins, showing maintained Loricrin (A; low risk, score = 0) and complete loss of Loricrin (B; high risk, score = 1) (m = melanoma, p = peri-tumoural epidermis, n = normal epidermis, scale bars = 100µm).

**Supplementary Figure 6. Relationship between Loricrin, and AMBRA1 and Loricrin expression in the Newcastle Discovery Cohort.**

AMBRA1 and Loricrin levels in the peritumoural epidermis of AJCC stage I melanomas were determined by pathologist visual inspection and defined as maintained or decreased. 80-month disease free survival rates were determined with the Kaplan-Meier method and compared by two-sided Log-Rank test for Loricrin analysis only (A; 20% vs 88.9%; P=0.026) or combined AMBRA1/Loricrin (B; 0% vs 100%; P<0.001).
